# Diagnostic Dilemma: IgG4-Related Sclerosing Mesenteritis Mimicking an Abdominal Malignancy Enveloping the Superior Mesenteric Artery

**DOI:** 10.7759/cureus.58480

**Published:** 2024-04-17

**Authors:** Kamran Ahmad, Mahnosh Saleh, Musa Kakakhel, Hasina Yasin, Yasir Ali, Hamid ur Rehman, Usama Saeed

**Affiliations:** 1 Internal Medicine, Hayatabad Medical Complex Peshawar, Peshawar, PAK; 2 Internal Medicine, Ayub Teaching Hospital, Abbottabad, PAK; 3 Stroke, Rehman Medical Institute, Peshawar, PAK; 4 Neurology, Rehman Medical Institute, Peshawar, PAK; 5 Urology, Medical Teaching Institute-Hayatabad Medical Complex Peshawar, Peshawar, PAK; 6 General Surgery, Medical Teaching Institute-Khyber Teaching Hospital, Peshawer, PAK

**Keywords:** surgical exploration, histopathological analysis, thalidomite responsive, immunoglobulin g4 serum levels, inflammatory disorder, igg4-related sclerosing mesenteritis superior mesenteric artery

## Abstract

Sclerosing mesenteritis, a rare fibroinflammatory disease affecting the mesentery, presents a diagnostic challenge due to its varied clinical manifestations and unknown etiology. We present a case of a 50-year-old female presenting with epigastric pain and weight loss, initially suspected of abdominal malignancy. Imaging revealed a mesenteric mass, and histopathological examination confirmed dense lymphoplasmacytic infiltrate with storiform fibrosis, along with elevated serum IgG4 levels, indicative of IgG4-related sclerosing mesenteritis. Treatment with thalidomide and prednisolone resulted in significant mass regression and symptom improvement. Our case highlights the importance of considering sclerosing mesenteritis in the differential diagnosis of abdominal masses and suggests a potential therapeutic approach for this rare condition. Further research is warranted to elucidate its pathogenesis and optimize management strategies.

## Introduction

Sclerosing mesenteritis is a spectrum of rare fibroinflammatory disease that affects the mesentery. It is characterized by the proliferation of fibrous tissue in the mesentery, ultimately compressing the surrounding organs. Mesenteric panniculitis was first described in 1924 by Jura et al. as "retractile mesenteritis" [1]. The name evolved over time when more information was revealed. Sclerosing mesenteritis is a broad term encompassing three similar entities based on their histology: mesenteric panniculitis, retractile mesenteritis, and mesenteric lipodystrophy [2]. These are either separate diseases or may represent different stages or manifestations of one disease, depending on the degree of inflammation, fatty necrosis, and fibrosis in the mesentery [2].

The cause of sclerosing mesenteritis is not well understood, but several pathological processes have been proposed, including abdominal surgery/trauma, autoimmune phenomena, paraneoplastic processes, and ischemia/infection. Abdominal surgery is frequently associated with the development of sclerosing mesenteritis, and autoimmunity may also play a role. The relationship between sclerosing mesenteritis and malignancy is debatable, with some studies showing a statistically significant association whereas others do not. Evidence of the role of infection in the development of sclerosing mesenteritis is limited. A better understanding of the pathogenesis of sclerosing mesenteritis may lead to improved diagnostic and management criteria [2]. Chronic inflammation and fibrosis can lead to various gastrointestinal complaints, such as abdominal pain, bloating, nausea/vomiting, weight loss, and fever [3]. When the histological and immunological features are associated with IgG4-related disease, the condition is defined as IgG4-related sclerosing mesenteritis [4]. The rare nature and complexity of sclerosing mesenteritis require further research to better understand its cause, pathogenesis, and optimal management strategies.

Here, we describe a case of IGg4-related sclerosing mesenteritis that mimicked abdominal malignancy.

## Case presentation

**Figure 1:**
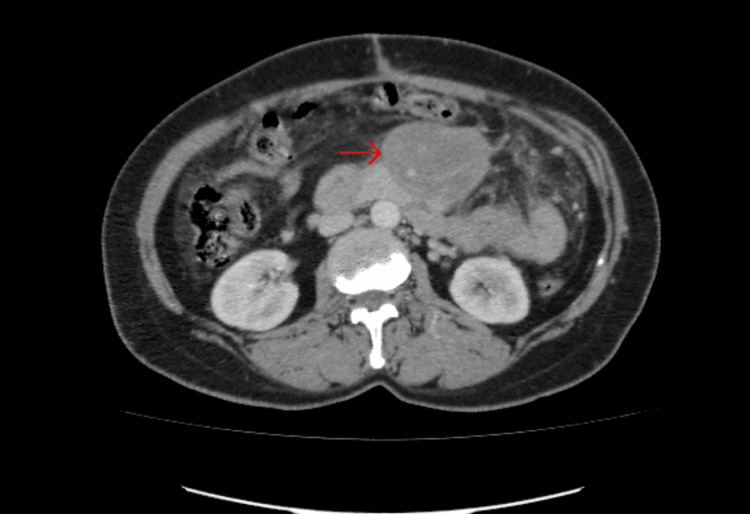
CT scan showing a mass encasing the mesenteric artery. A baseline CT scan shows a large mass. Red arrow pointing to the mass.

A 50-year-old female presented with a three-month history of epigastric pain that was dull in nature, non-radiating, postprandial, and relieved with over-the-counter analgesics. The pain was transient and worsened with increased mobility. The patient also reported a 10-kg weight loss over the same period, along with anorexia, but no vomiting. Upon examination, the epigastric mass was palpated. The mass was approximately closed fist size, non-tender, soft, mobile, and smooth with no jagged edges. No evidence of organomegaly or palpable lymphadenopathy was found. Baseline investigations were normal (Table 1), and ultrasound revealed a lobulated, avidly enhancing mesenteric mass around the superior mesenteric artery and its branches. A CT tomography was recommended (Figure 1), and a biopsy was advised for histopathology.

**Table 1:** Laboratory investigations with reference ranges.

Investigation	Results	Reference ranges
Total leukocyte count (TLC)	4900/mm^3^	4500–11,000/mm^3^
Hemoglobin (Hb)	10 g/dl	13.5–17.5 g/dL
Platelets (PLT)	311 x 10^9^/L	150–400 x 10^9^/L
Creatinine	0.9 mg/dL	0.66–1.25 mg/dL
Urea	38 mg/dL	5–20 mg/dl
Alanine amino transferase (ALT)	15 U/L	0–45 IU/L
Alkaline phosphatase (ALP)	20 U/L	0–35 IU/L
Aspartate aminotransferase (AST)	41 U/L	30–120 IU/L
Bilirubin	0.7 mg/dL	0.1–1.2 mg/dL
Sodium (Na)	136 mmol/L	136–144 mmol/L
Potassium (K)	2.7 mmol/L	3.7–5.1 mmol/L
Chloride (Cl)	107 mmol/L	98–107 mmol/L
Calcium (Ca)	9.3 mg/dL	8.5–10.2 mg/dL
Serum IgG4	1950 mg/dL	39–864 mg/dL
Serum CA 19-9	17.6 IU/mL	<37 U/mL
Serum CA 125	60.20 IU/mL	2–30 IU/mL

An ultrasound-guided biopsy revealed a circumferential mass around the superior mesenteric artery and its branches with dense chronic lymphocytic infiltrates. No well-formed granulomas or malignancies were observed. A repeat biopsy revealed atypical cells; however, the findings were inconclusive.

**Figure 2:**
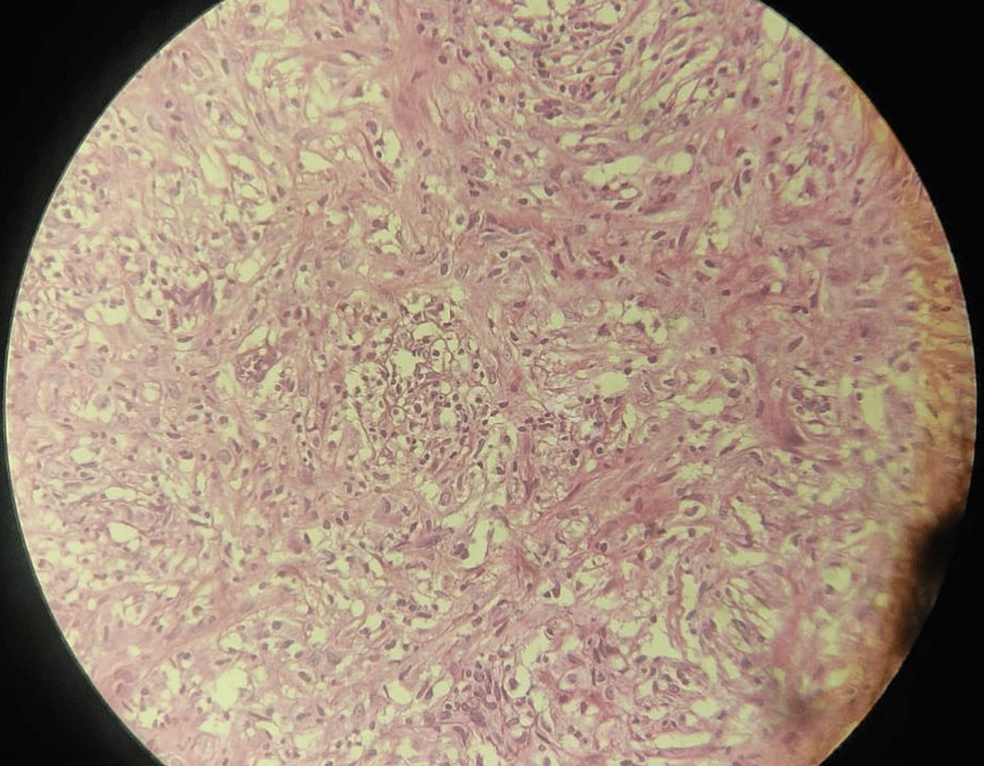
Plasma cells in the mesenteric mass. Biopsy sample of the mass found in the abdomen encasing the mesenteric artery.

**Figure 3:**
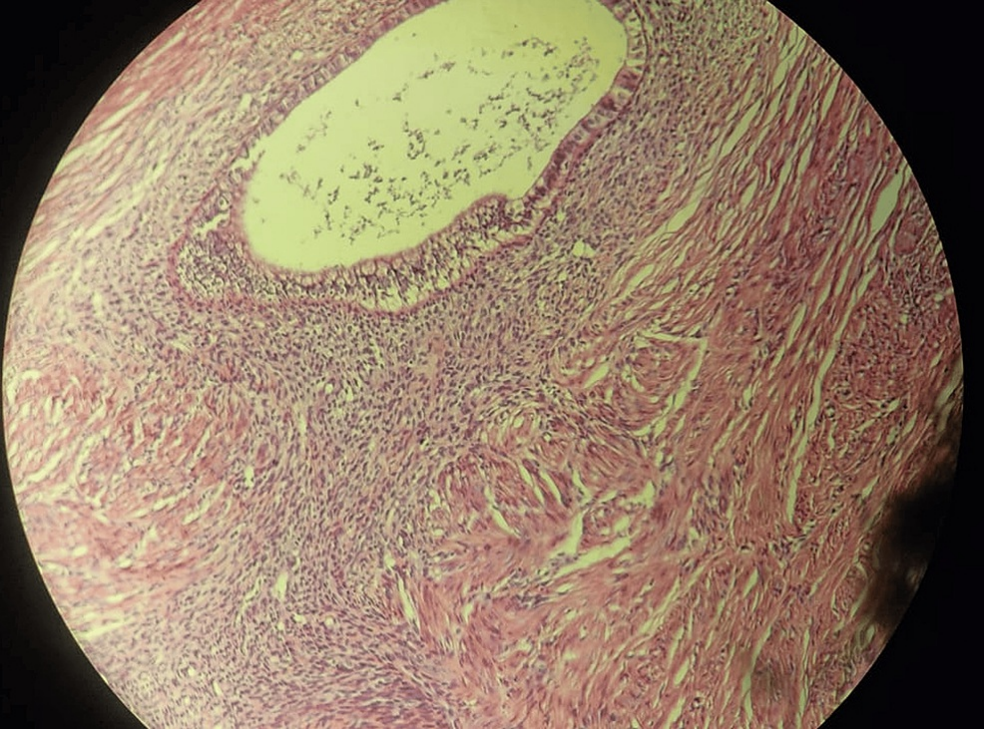
IgG4 positive plasma cells in the background of storiform fibrosis. A biopsy sample of the mass stained with IgG4.

Histopathological examination of the excisional biopsy revealed marked fibrosis with a storiform pattern, chronic lymphoplasmacytic inflammation, and an increased number of IgG4-positive plasma cells, suggesting the possibility of IgG4-related disease (sclerosing mesenteritis) (Figures 2-3).

The serum IgG4 level was elevated (1954 mg/L), and the patient was referred to an oncologist for further management. Thalidomide (100 mg) was prescribed once daily, along with prednisolone (40 mg) for two weeks and supportive care.

**Figure 4:**
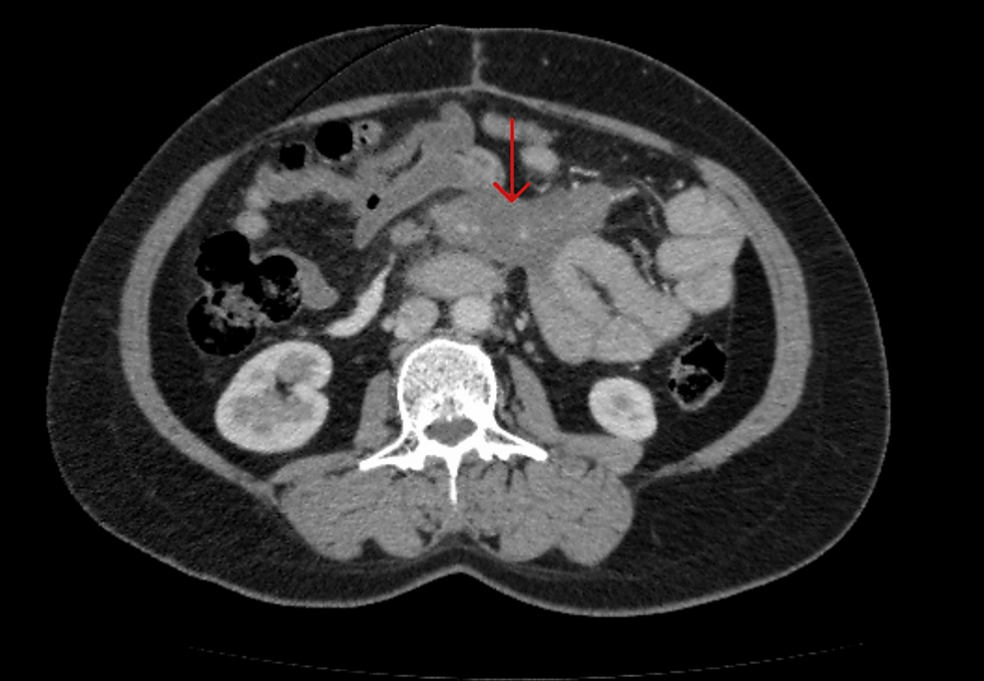
CT scan showing the mesenteric mass encasing the mesenteric artery. A CT scan at the six-month follow-up showing a mild regression in mass size, compared with Figure 1. Red arrow pointing to the mass.

**Figure 5:**
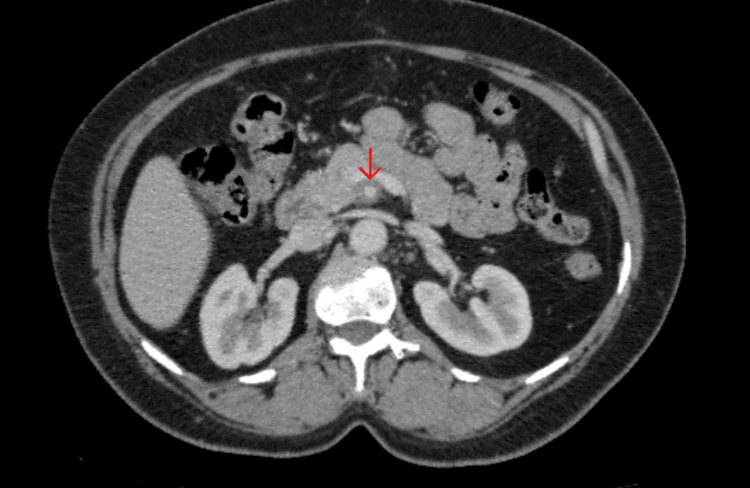
CT scan at nine month follow-up. The CT scan is showing an almost complete regression of the mass. Red arrow pointing to the mass.

After six months of follow-up, the patient reported improved appetite and regained her previous weight (Figure 4). A follow-up CT scan showed that the size of the mass had regressed by 20%. At nine months, follow-up computed tomography (CT) revealed a significant reduction in the size of the mass (Figure 5).

## Discussion

Our patient presented with chronic abdominal pain, which increased postprandial. She also experienced objective weight loss, which initially raised the suspicion of malignancy. However, the patient was later diagnosed with sclerosing mesenteritis. However, a rare disease has yet to be defined. It is a disease of the middle age or older decade of life and is twice as common in men as in women [2,3].

An excisional biopsy and histology of the sample taken from the mesenteric mass revealed dense lymphoplasmacytic infiltrate and storiform fibrosis, with certain areas of obliterative phlebitis on morphology. IgG4 staining showed an increased number of IgG4 cells in tissue with increased (1954) serum IgG4 levels, pointing toward the etiology of sclerosing mesenteritis as an IgG4-related disease in our patient. Based on the 2011 CDC criteria, it was updated in 2017 [5]. A consensus statement on the pathology of IgG4-related disease formed an inclusion criterion; the critical histopathological features were dense lymphoplasmacytic infiltrates [2], a storiform pattern of fibrosis [3], and obliterative phlebitis. While the tissue IgG4:IgG ratio is of secondary importance, Japan, where the disease is relatively more prevalent in the world, gives more weight to the IgG4:IgG ratio > 40%, as they believe there are other mimics of the disease, such as multicentric Castleman disease, rheumatoid arthritis, and other immune-mediated conditions that could present similarly [6].

IgG4-related disease is a newly recognized fibroinflammatory condition characterized by the above features; however, the areas of involvement classically involve the biliary tree, salivary glands, periorbital tissue, kidneys, lungs, lymph nodes, meninges, aorta, breast, prostate, thyroid, pericardium, and skin [7]. The mesentery is, however, not a very common area of isolated involvement, as an extensive search of the literature revealed only seven cases so far. Therefore, our case report adds to the list of rare diseases involving a rare location [5].

Sclerosing mesenteritis is less commonly caused by autoimmune etiology and more frequently associated with a history of abdominal surgery or trauma, malignancies or paraneoplastic phenomena, the autoimmune disease process, ischemia, and infection. Prior abdominal surgery ranges from 24% to 53% [2]. There is no consensus yet on the treatment of the disease. A treatment algorithm was proposed based on a case series of 92 patients [3]. Ranging from no treatment for asymptomatic patients to prednisolone and tamoxifen for nonobstructive symptoms, surgery is reserved only for complications and treatment-refractory cases.

Summary of the main findings of this study and implications for the diagnosis and management of sclerosing mesenteritis

Our patient was one of the rare cases with IgG4-related sclerosing mesenteritis and was treated with thalidomide and prednisolone. She responded well to treatment, and the mass showed size regression within six months. The patient's symptoms resolved completely, in accordance with the findings of previous case reports on the condition. In conclusion, while sclerosing mesenteritis may share certain features with IgG4-RD, it is likely to be a distinct entity and is not frequently associated with IgG4-RD.

## Conclusions

In conclusion, our case highlights the importance of recognizing sclerosing mesenteritis, particularly its IgG4-related variant, in patients presenting with abdominal symptoms and masses. The successful management of our patient with thalidomide and prednisolone suggests a promising therapeutic option for this condition. However, further research is needed to better understand the disease's underlying causes and to refine treatment protocols. By increasing awareness and understanding of sclerosing mesenteritis, we can improve diagnosis and enhance patient outcomes in the future.
